# Standardisation of bioacoustic terminology for insects

**DOI:** 10.3897/BDJ.8.e54222

**Published:** 2020-08-04

**Authors:** Edward Baker, David Chesmore

**Affiliations:** 1 University of York, York, United Kingdom University of York York United Kingdom

**Keywords:** insect, sound production, vocabulary, bioacoustics

## Abstract

After reviewing the published literature on sound production in insects, a standardised terminology and controlled vocabularies have been created. This combined terminology has potential for use in automated identification systems, evolutionary studies, and other use cases where the synthesis of bioacoustic traits from the literature is required. An example implementation has been developed for the BioAcoustica platform. It is hoped that future development of controlled vocabularies will become a community effort.

## Introduction

"*Two dangers face the student seeking to rationalize and codify a terminology that has grown up empirically and that is beginning to differentiate regionally or according to faculty or in other ways - as must always tend to happen. One danger is that of legislating prematurely and clumsily for hypothetical future requirements; the other is a too easy-going and long-sustained attitude of laissez-faire arising from wishing to let the mud settle before trying to penetrate the shadows of often chaotic and obscure usages. If the former danger must always be borne in mind, the latter is more insidious; while we wait for the mud to settle, divergence may be increasing, and we may be faced with the need to cure what we might have prevented.*" - Broughton (1963)

The stereotypical songs of the singing insects (particularly Orthoptera and Hemiptera: Cicadidae) have been used to describe species (Heller and Baker 2017), undertake population surveys (Brock 2017) and to estimate biodiversity (Sueur et al. 2014). While these are the best-known of the audible insects, many other species can produce sound, and examples are found in orders including Lepidoptera (Brehm et al. 2015, Travassos and Pierce 2000), Diptera (Sueur et al. 2005, Cator et al. 2009), Coleoptera (Lyal and King 1996, Buchler et al. 1981), Phasmida (Henry 1922, Bragg 1992), Blattodea (Hunsinger et al. 2018) and Neuroptera (Price et al. 2015). The acoustic behaviour of the Orthoptera has been comprehensively reviewed (Robinson and Hall 2002), and although these authors noted the lack of conformity in structural descriptions of songs, they did not suggest a solution to this issue.

Several acoustic libraries have significant volumes of insect recordings, such as BioAcoustica (Baker et al. 2015b) which contains the Library of Recorded Insect Sounds from the Natural History Museum, London as well as contributions from numerous other individuals. A list of sound archives with significant Orthoptera holdings is given in Riede 2018. The Global Cicada Sound Collection is a project to collate worldwide cicada sound collections within BioAcoustica (Baker et al. 2015a, Baker 2016a). In addition a large amount of literature has been published on the acoustics of insects, but often without deposition of accompanying recordings (Baker and Vincent 2019).

Information about the sounds produced by insects is essential for work on automated acoustic monitoring (e.g. Bennet et al. 2015) and taxonomy (e.g. Ragge 1990). Large scale studies need to synthesise data both from published literature and from analysis of recorded sounds. Automated extraction of acoustic characters from recordings is becoming increasingly feasible (Riede et al. 2006) and increasingly desirable with large scale acoustic monitoring becoming more common (Truskinger et al. 2018, Sethi et al. 2018). Insects are a prime, though underused, candidate for automated identification: "A rigid determinism governs, in most cases, sound production among arthropods" (Dumortier 1963).

Despite the plentiful data from recordings and published works, comparison of species across these datasets is complicated by the lack of a single terminology. This work proposes a formalised terminology for describing insect song, as well as controlled vocabularies for types of call and methods of sound production. Together these components can be used to collate published acoustic traits from the literature and analyses performed on sound libraries, as well as providing a clear and concise framework for publishing and sharing new findings. While at present limited to the deliberate production of sound by insects, the terminology and vocabularies are openly published and so may be extended to other taxonomic groups by future researchers.

Automated identification of species using acoustics is the aim of several projects (e.g. the New Forest Cicada Project: http://www.newforestcicada.info). The accuracy of such systems could be improved with knowledge not just of the calls themselves, but the environmental and temporal conditions that may influence the calls. For this reason, this terminology allows the recording of properties such as the minimum environmental temperature at which a species will produce a call, and temporal (daily and yearly) calling patterns.

Methods for integrating this terminology with others, such as DarwinCore (Wieczorek et al. 2012) are suggested. DarwinCore archives are already used to link multiple data providers to global aggregators such as the Global Biodiversity Informatics Facility and the Encyclopedia of Life (Baker et al. 2014), and some sound collections already use DarwinCore archives to share their data (e.g. Baker et al. 2015a).

### Example use cases

**Acoustic Keys**

Many authors provide keys to acoustic identification of small groups of insect species in their papers (REF), and there are a smaller number of comprehensive regional identification keys (e.g. Ragge and Reynolds 1998). A comprehensive database of acoustic traits would allow for automated generation of dichotomous or matrix-based keys. The increased accessibility of species distribution data via GBIF, combined with terms proposed here recording the time of year and time of day of calls, would allow for the automatic generation of keys that are both geographically localised and temporally relevant.

**Automated identification**

While there are many large datasets available for bird song (see for example those used in the Bird Audio Detection Challenge: Stowell et al. 2016) there are no such comparably large datasets for insect sounds. Many studies of machine learning methods in insects, by necessity, use datasets that are orders of magnitude smaller in size (e.g. Chesmore and Ohya 2007). Therefore while the reliable classification of broad categories of insect song should be possible with machine learning methods, reliable identification of species beyond a small taxonomic or geographic scope is not. Machine-readable datasets of sound parameters may, therefore, provide a useful intermediate, particularly when combined with other datasets. For well-studied orthopteran faunas, such as the United Kingdom, many species can be distinguished solely on the peak frequency of their song. A route to a reliable automated identification system may, therefore, be a hierarchical classifier where the identification of 'Orthoptera' is made by machine learning, and a database of known acoustic traits is used to provide a species identification. Combined with other datasets (e.g. distribution, habitat, phenology) such identifications could be further refined.

**Evolution of song**

Combined with an appropriate phylogeny, well defined acoustic traits could be easily used to make inferences about the evolution of sound production. A number of previous studies have used acoustic traits to study evolution (e.g. Robillard et al. 2007, Nattier et al. 2011). The creation of a database of traits would make the data collection for such studies easier.

## Material and methods

While collecting literature data about the songs of Orthoptera, the terminologies used to describe song structure and traits were collected. In order to allow comparison between terminologies a formalised vocabulary was developed that eliminates synonymous terms and allows for suitable levels of precision to be identified (e.g. differentiating between ’peak frequency’ and ’frequency range’).

This paper describes the terms used in the description voabulary as well as documenting the decisions made when choosing between alternative representations and terms.

**Units**

Units for each proposed term are generally SI units unless prevailing usage is otherwise. Units are only given in the text when SI units are not proposed.

## "Bag of terms": ontology or vocabulary

The creation of a formal ontology for describing insect song was rejected by the authors, despite the potential personal intellectual reward for doing so. Instead, the scheme proposed here is a set of defined terms used to describe insect song, as well as some proposed lists of values (controlled vocabularies). This "bag of terms" approach has seen success in the development of DarwinCore (Wieczorek et al. 2012) and other related systems such as AudubonCore (Morris et al. 2013).

With the aim of future community involvement in the development of this vocabulary, and with the authors having watched closely the development of DarwinCore this approach appears to give the most flexibility. Much has been written on the development of standards, and this quote is one of many that could summarise the approach taken here: "*Notice I said 'vocabulary' and not 'ontology'. The less ontology there is in the shared Core, the easier it will be for people to build on it to suit their needs. But a lack of ontology does not imply a lack of semantics*" (Sachs 2013).

## Data resources

The ontology and controlled vocabularies are presented here, and are available online at https://vocab.audioblast.org.

It is hoped that other interested parties will become involved in the development of the ontology. Contributions can be made via the project’s GitHub page at https://github.com/audioblast/vocabularies.

## Results

The terms and controlled vocabularies developed are presented here in categories. An alphabetical list of terms is available at https://vocab.audioblast.org. Terms in the text are followed by their identifier Uniform Resource Identifier (URI); terms in the tables are hyperlinked to the URI.

### Types of call

Presented is a controlled vocabulary (Table 1) of the different call types produced by insects. Synonymous terms are presented in the table, and definitions are provided below. Only actively produced sounds are listed (i.e. those that are deliberately produced and have a biological function, and also involuntary sounds produced by the organism such as flight buzzes). Passive sounds, such as scuttling or rustling of the substrate, have been excluded at this stage.

**Types of call and their function(s)**

While this controlled vocabulary is for call type, a possible use case is to compare calls with the same or similar function. Some gomphocerine grasshoppers, for example, have multiple distinct types of call between the successful attraction of a mate and mating. These call types can be grouped together using a higher-level term (in this case PrematingSong) to facilitate analysis by call function.

**CallType** http://vocab.audioblast.org/CallType

This term is used to specify a type of call or song, recommended practise is to use the controlled vocabulary presented here.

#### Calling Song

The calling song is produced by a male in order to attract a female (in species which also have a separate song for courtship the calling song is used to bring a pair together before the courtship rituals). Multiple males may join together to form a chorus, either synchronising or alternating their calling songs. This is the most commonly produced sound by male orthopterans and cicadas.

#### Response Song

Female response to the male's call during the mate-attraction phase (i.e. male-female duets for phonotaxis).

#### Congregating Song

Dumortier (1963) discusses differences between the congregating song and the calling song: "the congregational song does not only attract the opposite sex whereas the calling song does. The congregational song produces the grouping of males, females or larvae."

#### Courtship Song

A special courtship song may be produced by the male when in close proximity to the female. Along with Response Song considered a 'Premating Song' by Dumortier (1963).

#### Agreement Song

The female’s response to the male song when she is receptive to mating and at close proximity. This is rarely heard in the field, but unmated females in the laboratory may sing spontaneously (Ragge and Reynolds 1998). Along with Courtship Song and Jumping Song considered a 'Premating Song' by Dumortier (1963).

#### Jumping Song

Characteristic of the Orthoptera: Acridinae, stridulation produced directly before the male mounts the female.

#### Post-copulatory Call

This post-mating call may function in mate-guarding and is present in some genera of the Gryllidae (Robinson and Hall 2002).

#### Rivalry Song

The calling song of the male may attract other males, and when in close proximity they may produce a modified song known as a rivalry song - often faster or abbreviated versions of the calling song (Ragge and Reynolds 1998).

#### Defensive Call

A call made to deter against perceived threats. The bush cricket *Anyclecha fenestrata* has defensive calls in both sexes (Greven et al. 2013) as do representatives of the beetle family Lamiinae (Finn et al. 1972).

#### Flight Noise

A distinction is made between ’Flight Noise’ as the ’buzzing’ sound made by many insects during any flight due to the movement of the wings, and crepitation where the sound is made by a different method. Crepitation in some species is facultative (occurring only in special display flights) whereas in others it occurs in all flights. Flight Noise is considered to be a type of call in some species (e.g. the mosquito *Aedes aegypti* (Linnaeus 1762) described in Cator et al. 2009), whereas crepitation is a method of sound production that functions as a Calling Song in many species.

### Sound Production Method

The classification of sound production mechanisms has been addressed by a number of previous authors. Ewing (1989) devised a categorisation based entirely on the physical mechanism of sound production (percussion, air expulsion, vibration, tymbal mechanisms and stridulation). Most insect sounds can be neatly placed into these categories, with the possible exception of crepitation. Crepitation, a snapping sound made by the wings, may be considered to be a form of tymbalisation, albeit not always under direct muscular control as it may be a by-product of flight. A broad interpretation of tymbalisation would include the crepitation of the Orthoptera. Crepitation is here retained as a separate term, but may in the broadest sense be treated as synonymous with tymbalisation.

The air expulsion of Ewing (1989) is here expanded to fluid expulsion, in recognition of the fact many insects are aquatic for at least part of their lives, and while freshwater acoustic studies of insects are presently limited, noise created by the expulsion of water would be analogous with the expulsion of air in terrestrial environments.

For each of these broad categories, a number of different body parts have evolved to become the apparatus of sound production. These are considered as subcategories of the main methods. Table 2 gives a controlled vocabulary of sound production mechanisms.

#### Stridulation

Stridulation has evolved multiple times within the insects, and further mechanisms may be discovered. The controlled vocabulary for Sound Production Method (Table 2) contains separate entries for each type of stridulation known.

In some cases distinction needs to be made between which of the two body parts has the file. Following Wessel (2006) the part which has the file (pars stridens) is given first, so there is a distinction made between Abdomino-alary and Alary-abdominal methods.

**StridulationInFlight** https://vocab.audioblast.org/StridulationInFlight

The bush crickets *Oxyecous lesnei* and *Debrona cervina* are able to stridulate in flight (Naskrecki and Guta 2019). Recommended values are 'Present', 'Absent'.

#### Vibration and Tremulation

Vibratory motions are classified into two types. Those where vibration of the body (or part thereof) transmits an acoustic signal through a fluid (air or water) are considered vibrations. Those where vibration is transmitted through a solid substrate, such as vegetation, are termed tremulation.

#### Tymbalisation

In most cicadas, sound production is primarily through the process of tymbalisation: the de-formation of the paired tymbals at a high rate. In cicadas, the tymbals are modified sections of abdominal tegumen strengthened by ridges that can be deformed by muscles (Pringle 1954).

#### Crepitation

Crepitation is a noise made by the snapping of wings as they extend, sometimes occurring facultatively as part of a special crepitation display flight, otherwise obligate and occurs in all flights.

A second definition is the sharp sound produced by rapid fluid discharge, e.g. in bombardier beetles (Gordh and Headrick 2001), although not for the hissing sound made by hissing cockroaches which is a rapid discharge of air through modified spiracles. Given the etymology comes from the Latin *crepito* suggesting a crackling sound reserving the definition to the first given seems logical. The second definition is covered in this vocabulary under FluidExpulsion.

#### Fluid Expulsion

The forced expulsion of air through modified spiracles creates the distinctive hiss in the hissing cockroaches (Blattodea: Blaberidae: Gromphadorhini; Hunsinger et al. 2018). The hawkmoth *Acherontia sphinx* makes a defensive sound by passing air through the pharynx (Brehm et al. 2015).

#### Percussion

Percussive noises are generated by the impact between body parts, or between part of the body and the substrate. Ewing (1989) notes that the exoskeleton of arthropods makes percussion an efficient communication method.

Moths of the genus *Hecatesia* have hardened sections of the fore wing called castanets that strike together in flight to produce sound, leading to their common name of 'whistling moths' Bailey (1978).

### Sound Propagation

**SoundPropagationMedium** https://vocab.audioblast.org/SoundPropagationMedium

The medium through which the sound propagates. A controlled vocabulary is provided (https://vocab.audioblast.org/cv/medium) with values 'air', 'freshwater' and 'substrate'. This vocabulary is open to expansion, particularly in more precise terms for varying substrates.

**SoundPropagationDistance** https://vocab.audioblast.org/SoundPropagationDistance

The literature contains many references to the distance at which insect sound remains perceptible to the human ear. While this information is of considerable use to the field naturalist, for rigorous acoustic analysis it is recommended that more precise definitions are defined in future.

### Descriptions of call structure

#### Syllables

The Orthoptera are the best known stridulatory organisms and are the focus of most attempts at describing biological stridulation. The terminology used by European (following, e.g. Broughton 1976, Ragge and Reynolds 1998) and North American workers (following, e.g. Walker and Dew 1972) is divergent although broadly the terms can be reconciled. The use of the term syllable to refer to a single complete stridulatory movement (the opening and closing of the elytra in Ensifera, the up and down motion of the femora against the elytra in some Acrididae) is supported by Ragge and Reynolds (1998) as the basic unit of stridulatory calls due to its precise biological definition. The definition is expanded to include diplosyllables (e.g. distinct opening and closing stridulation of the elytra in some Ensifera) and hemisyllables (where only one of these motions produces sound). Such terminology can easily be expanded to many other stridulatory mechanisms, and may also be expanded to other sound production methods involving a to-and-fro movement such as tymbalisation.

Each (hemi-)syllable is comprised of one or more tooth impacts. While each tooth impact can produce a pulse of sound, the terminology of pulses and pulse trains is inconsistent amongst workers (in particular Cole 2010). While tooth impacts have a biological meaning related to the stridulatory structures, there is a possibility that rapid impacts in succession may not be acoustically resolved at a distance, particularly if the sound-producing apparatus are highly resonant. The term pulse as used in other bioacoustics fields (e.g. anurans Köhler et al. 2017) to describe an indivisible unit of sound seems appropriate for use as the most basic unit of stridulatory sounds, although the term does come with with "epistemological problems" (Appleby 1987): "Pulse is surely the most ill-used term ever taken over by the bio-acoustician" (Broughton 1963).

**SyllableGapNumber** https://vocab.audioblast.org/SyllableGapNumber

Identifying the number of silent periods, or gaps, within a syllable can be diagnostic to some species of Orthoptera (Ragge and Reynolds 1998).

#### Echemes and Echeme-Sequences

While Broughton (1976) replaced the term ’chirp’ with ’echeme’, there are additional terminologies that have been applied to what is considered here to be an echeme. Sakaguchi and Gray (2011) touch on this confusion between chirps and trills in crickets of the genus *Gryllus*, while introducing a new term 'stutter-trill'. While such terms may be of use in casual descriptions of songs, and indeed do convey meaning (particularly for human identification by ear), they are not useful in a rigorous analysis without being decomposed into a standardised terminology. Both chirps and trills are a first-order assemblage of syllables, and are therefore echemes differing in their number of syllables.

Similarly, the term 'bout' as used by Hedrick (1986) and others is an echeme-sequence (a first-order assemblage of echemes).

For convenience, an echeme-sequence may include syllables that are produced in association with an echeme, e.g. the song of *Arcyptera fascia* consists of a dense echeme preceded and followed by individual syllables.

#### Interval, duration and spacing

Various authors use different terms for describing the space between elements of a song. The gap between syllables may various take the form of syllable spacing, syllable interval and ’intersyllable duration’. The terms adopted here are illustrated in Fig. 2.

#### Standard Descriptive Units

Various terms are used to describe individual components of insect song in the published literature. While they are not strictly needed by the method for describing songs using this ontology, the inclusion of terms that have a defined meaning is useful (e.g. comparison of echeme length in a group of related species, or with temperature). The controlled vocabulary in Table 3 is proposed. Figure 2 provides an outline of the major components (syllable, echeme and echeme sequence), the extra terms in the table are modifications of these basic structures.

**Wing-beatFrequency** https://vocab.audioblast.org/Wing-beatFrequency

The frequency at which the wings beat during flight producing a 'buzz' noise.

**CallStructure** https://vocab.audioblast.org/CallStructure

Highest unit of call structure, e.g. 'Syllable' or 'Echeme Sequence'.

**CrepitationRate** https://vocab.audioblast.org/CrepitationRate

The number of crepitation sounds made per second (Hz).

**CrepitationDuration** https://vocab.audioblast.org/CrepitationDuration

The duration of one crepitation sound.

**CrepitationInterval** https://vocab.audioblast.org/CrepitationInterval

The time between individual crepitation sounds.

**CrepitationIsFaculative** https://vocab.audioblast.org/CrepitationIsFaculative

'True' or 'False'. In some species, crepitation is controlled and only used in crepitation displays; in others it is uncontrolled and occurs during any flight (Ragge and Reynolds 1998).

**PercussionImpactRate** https://vocab.audioblast.org/PercussionImpactRate

The number of percussive impacts per second (Hz).

**PercussionImpactsPerCall** https://vocab.audioblast.org/PercussionImpactsPerCall

### Call Properties

#### Amplitude

https://vocab.audioblast.org/AmplitudeUnit: dB

While the concept of call amplitude is easily understood, it can be measured in a wide variety of ways. The distance from the subject is of clear importance. The property ’Amplitude’ has been included in the ontology, however, it is hoped that more specific sub-properties can be agreed upon in the future. These should include a standardised unit of measure and distance from the subject.

**AmplitudeWithBaffle**: https://vocab.audioblast.org/AmplitudeWithBaffle

A baffle may be used to amplify the song (see below, External resonators).

#### Frequency

https://vocab.audioblast.org/Frequency

In published works, the method of calculating the frequency or frequency range is not always given. The sub-properties of this property allow for precise definitions to be attributed where possible.

**FundamentalFrequency** https://vocab.audioblast.org/FundamentalFrequency

**PeakFrequency** https://vocab.audioblast.org/PeakFrequency

This is the frequency with the highest amplitude. It is often the same as the fundamental frequency in resonant songs, however, the resonators may make one of the harmonics have a greater amplitude than the fundamental.

**Bandwidth** https://vocab.audioblast.org/Bandwidth

**Figure 1.**
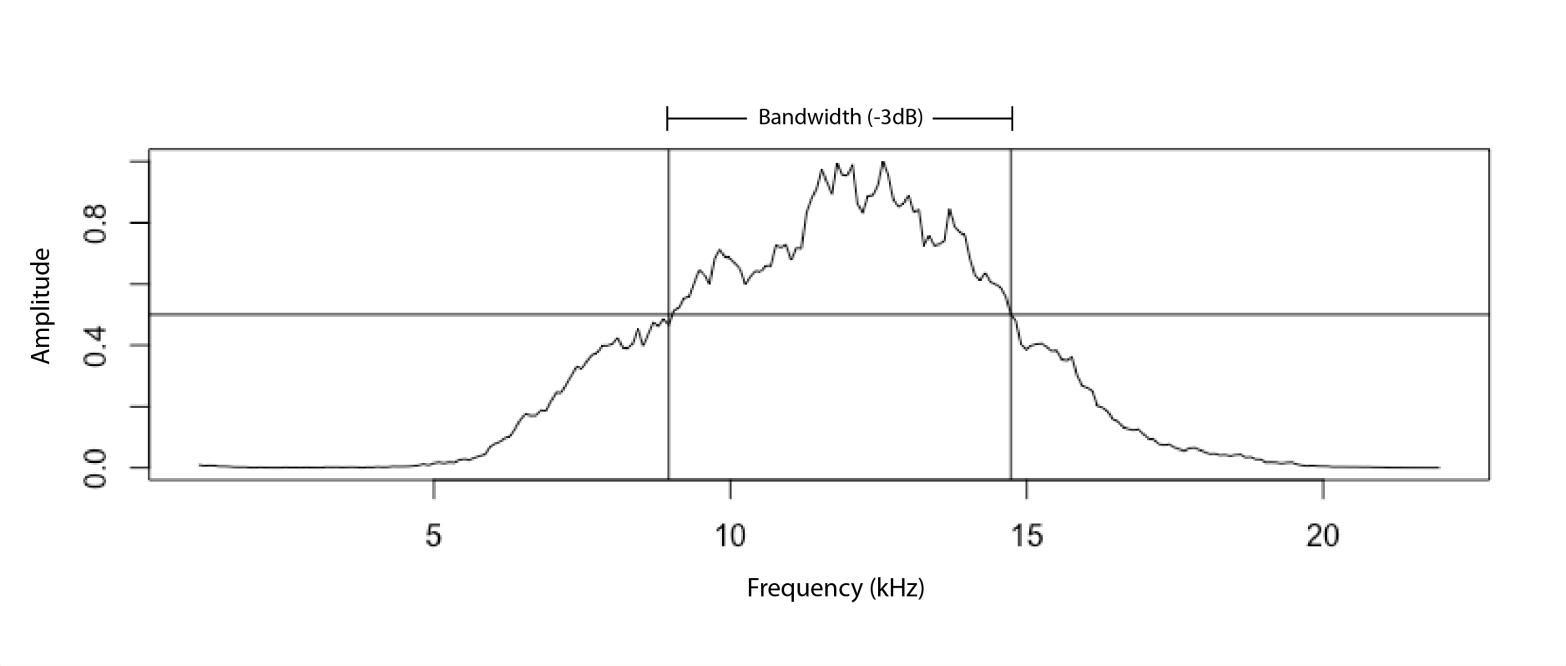
The -3dB bandwidth.

**Figure 2.**
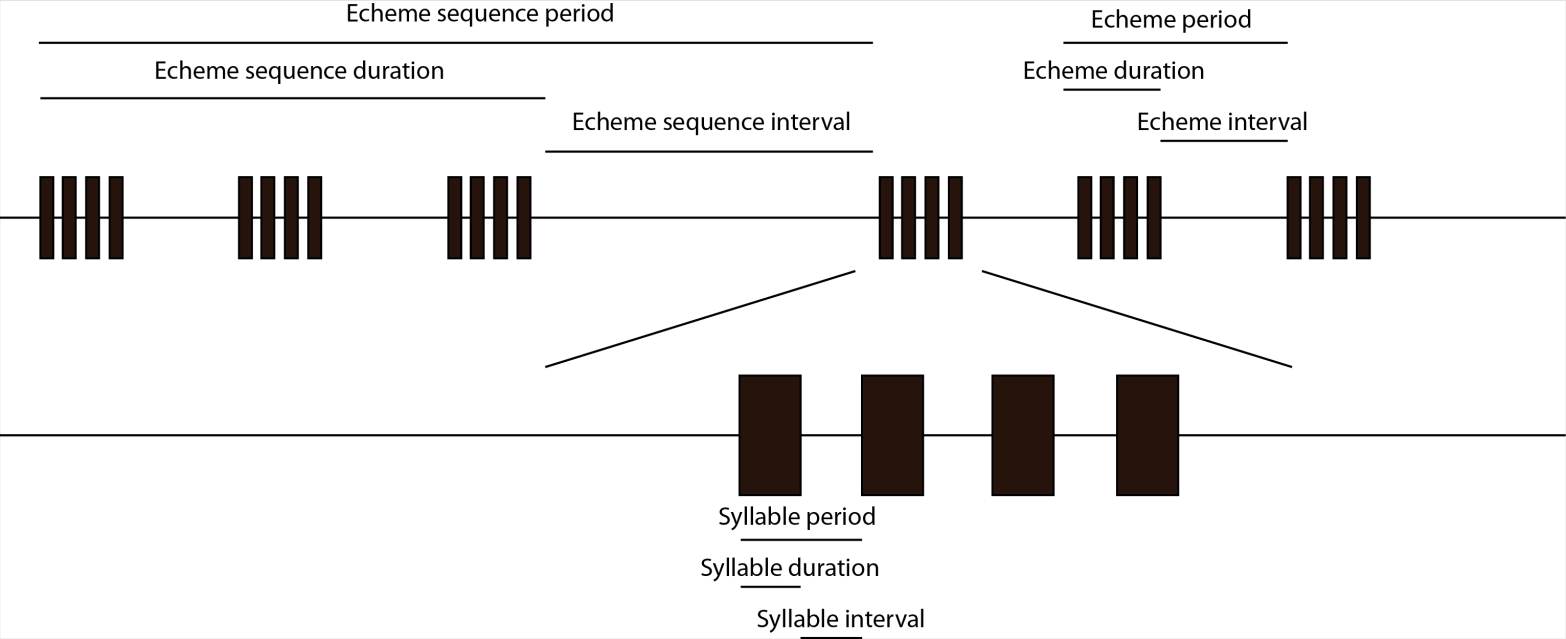
Relationship of period, duration and interval.

The bandwidth is usually defined as the range of frequencies around the peak frequency with an amplitude greater than half (-3dB) of the peak frequency (Fig. 1), although -10dB may also be used, for discussion see Bennet-Clark (1999).

**Bandwidth -10dB** https://vocab.audioblast.org/Bandwidth-10dB

**CentreFrequency** https://vocab.audioblast.org/CentreFrequency

This is the middle point of the bandwidth.

**Q-factor** https://vocab.audioblast.org/Qfactor

The Q-factor (quality factor) is the ratio of the resonant frequency of a system to the bandwidth at which the power is over half of the maximum (-3dB). Other methods of calculating Q exist (Bennet-Clark 1999). In the case of cricket wings, these have shown to be similar (Nocke 1971).

The distinction between Q and Q_10_dB has previously caused confusion in the bioacoustics literature (Bennet-Clark 1999). Outside of bioacoustics Q is generally calculated with a -3dB bandwidth as defined here.

**DominantHarmonic** https://vocab.audioblast.org/DominantHarmonic

The harmonic with the largest amplitude (1st, 2nd, etc.)

**FirstHarmonicFrequency** https://vocab.audioblast.org/FirstHarmonicFrequency

The frequency of the first harmonic, in kHz.

**FirstHarmonicAttenuation** https://vocab.audioblast.org/FirstHarmonicAttenuation

The difference in amplitude between the fundamental and first harmonic amplitude (dB).

**SecondHarmonicFrequency** https://vocab.audioblast.org/SecondHarmonicFrequency

The frequency of the second harmonic, in kHz.

**SecondHarmonicAttenuation** https://vocab.audioblast.org/SecondHarmonicAttenuation

The difference in amplitude between the fundamental and second harmonic amplitude (dB).

#### Duty Cycle

https://vocab.audioblast.org/DutyCycle

The duty cycle is the percentage of a cycle for which a signal is present. When the song has a higher-order structure (e.g. echemes), there will be multiple duty cycles (e.g. for syllables within an echeme and for the entire song).

### Calling Conditions

#### Temporal

While some species will sing throughout the day and night, others make their Calling Songs mostly, or only, at certain times of the day. The data property time of day of call allows these data to be recorded. While some literature gives the timing in hours (in which case it should be recorded as, e.g. 1100-1500) others use terms such as ’late afternoon’ or ’evening’. While it may appear that giving actual times may be more precise than these looser terms, that may not always be the case. The timing of evening as an example will vary both with latitude and potentially the time of year. In the case of an automated recognition system that is aware of both its time and location, and can, therefore, calculate when it is likely to be evening on any given day, the looser time may provide a more helpful hint at identification. In addition to diel patterns in Calling Song, there may also be yearly cycles in call production, particularly in temperate regions. The time of year of call property allows this to be recorded (e.g. Late June-September).

**TimeOfDayOfCall** https://vocab.audioblast.org/TimeOfDayOfCall

**TimeOfDayOfHighestAcousticActivity** https://vocab.audioblast.org/TimeOfDayOfHighestAcousticActivity

**TimeofYearOfCall** https://vocab.audioblast.org/TimeOfYearOfCall

#### Environmental

**MinimumCallingTemperature** https://vocab.audioblast.org/MinimumCallingTemperature

Many species will not produce a calling song below a particular temperature (e.g. *Ephippiger ephippiger* will not stridulate below 15-17^o^C (Stiedl and Bickmeyer 1991).

**CallingHeight** https://vocab.audioblast.org/CallingHeight

Many insects call from a specific height within the environment.

### Call Participants

#### Male-female duets

In most species, the male calls and the female remains silent while approaching her potential mate. However, in a few groups of Orthoptera and Cicadidae, the female signals acoustically to the male, who may modify his call rate in response. This female Response Song occurs during the mate location stage and is therefore different from the Agreement Song, which occurs when the male and female are within close proximity. Response songs are currently only known from three unrelated lineages in the Tettigoniidae (Robinson and Hall 2002) and some cicadas.

In some species the female moves towards the male (female phonotaxis), in others the male towards the female (male phonotaxis). In other species, the male and/or female will perform phonotaxis. The recommended values for the mating location method data property are given in Table 4.

**FemaleResponseDelay** https://vocab.audioblast.org/FemaleResponseDelay

Some species have a very narrow window in which the female must reply to maintain phonotaxis, notably the common European species *Leptophyes punctatissima* has a response window of only 20-50ms (Robinson and Hall 2002). Similar female responses that are dependant on signal timing are found in some cicada species (Marshall and Cooley 2001). The data property female response window can be used to store this data, although there are few studies in the literature.

**CallParticipants** https://vocab.audioblast.org/CallParticipants

One of 'Male', 'Female', 'MaleAndFemale'.

#### Male response to male Calling Song

The presence of a conspecific Calling Song may change the acoustic behaviour of a male. A controlled vocabulary of these behaviour modifications is given in Table 5.

**Physical spacing** The Calling Song of a conspecific male may be an agonistic signal. The reaction of males to conspecific Calling Songs can vary, some such as *Tettigonia viridissima* try to maximise their distance from other males (Physical Spacing) (Arak et al. 1990) (but the spacing may be limited by habitat features, such as suitable singing perches: Arak and Eiriksson 1992). Species that sing at the same time of day but do not modify their acoustic behaviour in response to conspecific song should not be included (e.g. those species which sing at dusk each evening).

**IndividualSpacingWhileCalling** https://vocab.audioblast.org/IndividualSpacingWhileCalling

**Figure 3.**
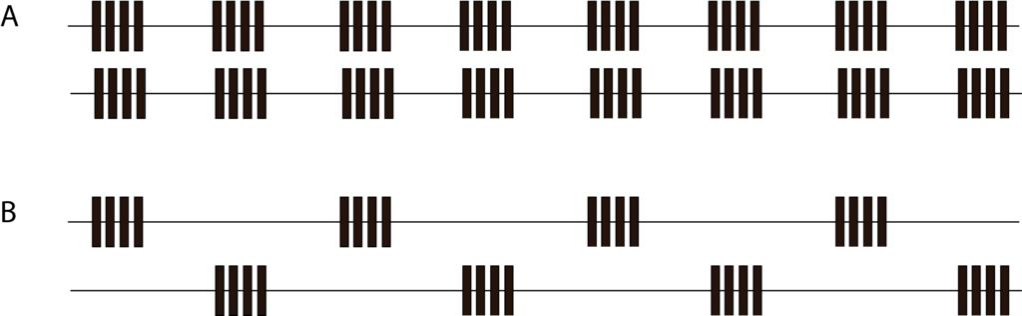
A: Synchronous Chorusing; B: Alternating Chorusing

**Chorusing** In Synchronous Chorusing conspecific males synchronise their songs to begin almost simultaneously. In Alternating Chorusing males (such as *Pterophylla camellifolia*; Shaw 1968) do not overlap the repeating units of their song. In both types of chorusing, the rhythm of the song may be more uniformly periodic than the same male singing in isolation. The different types of chorusing are shown in Fig. 3.

Unsychronous chorusing occurs when groups of individuals produce a call, but no relationship appears to occur between the calls of individuals (Ewing 1989).

Chorusing males may sing more frequently and more often than solitary males of the same species (Alexander 1967).

#### Alternatives to acoustic communication

**AlternateMateAttractionMethod** https://vocab.audioblast.org/AlternateMateAttractionMethod

Often acosutic signalling is combined with other signalling methods, such as 'Visual'.

### Sound production morphology

#### Stridulatory apparatus

**Figure 4.**
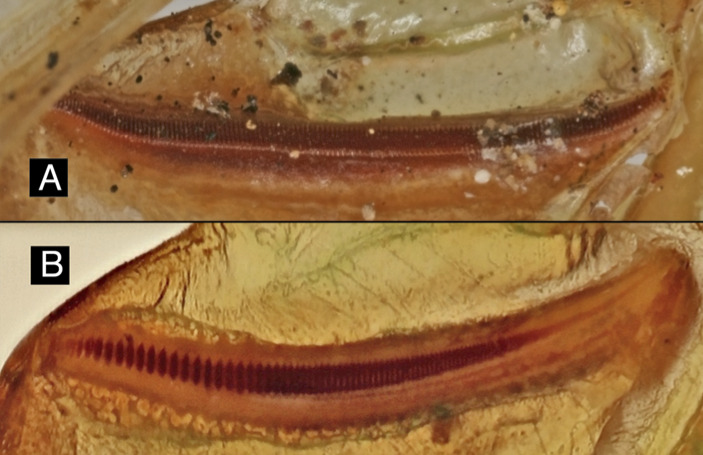
The stridulatory files of two closely related species of *Horatosphaga* (Heller and Baker 2017).

A stridulatory apparatus consists of a plectrum (often a raised vein on a wing) and a file, a series of raised protrusions. The stridulatory files of two closely related species of bush cricket are shown in Fig. 4, demonstrating the variation in stimulatory apparatus even within a single genus.

Both the length of the stridulatory file and the number of teeth on the file can be diagnostic to species and are included in this ontology.

**StridulatoryFileLength** https://vocab.audioblast.org/StridulatoryFileLength

Unit:mm

**StridulatoryFileToothNumber** https://vocab.audioblast.org/StridulatoryFileToothNumber

**StridulatoryFileToothDensity** https://vocab.audioblast.org/StridulatoryFileToothDensity

Unit: teeth per mm

**StridulatoryFileWidth** https://vocab.audioblast.org/StridulatoryFileWidth

Unit: mm

**StridulatoryFileToothWidth** https://vocab.audioblast.org/StridulatoryFileToothWidth

Unit: μm

**StridulatoryFileImpactsPerSyllable** https://vocab.audioblast.org/StridulatoryFileImpactsPerSyllable

#### Tymbalisation apparatus

The tymablisation apparatus consists of a rigid membrane that produces sound as it is buckled. The sound produced may be altered by the presence of ribs that cause the deformation to happen in disticnt stages.

**TymablRibNumber** https://vocab.audioblast.org/TymbalRibNumber

#### Resonators

https://vocab.audioblast.org/Resonator

Resonators are often used to tune and amplify the songs of insects. Multiple resonators may be used, such as the 'harp' and 'mirror' in crickets.

**PrimaryResonator** https://vocab.audioblast.org/PrimaryResonator

**SecondaryResonator** https://vocab.audioblast.org/SecondaryResonator

### External resonators

#### Acoustic burrows

**Figure 5.**
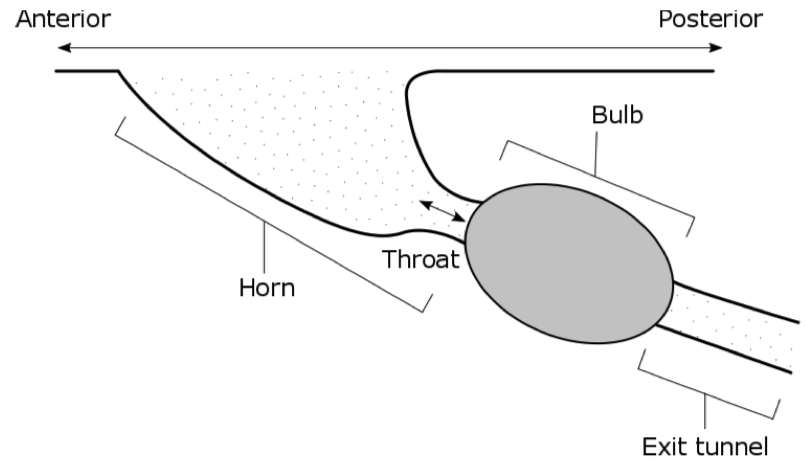
Acoustic burrow of *Gryllotalpa major* from Baker (2016b).

Various species of Orthoptera use burrows as external resonators to amplify their calls (Fig. 5), this behaviour is most obvious in the mole crickets (Orthoptera: Gryllotalpidae). The acoustic properties of acoustic burrows have been discussed by Bennet-Clark, a descriptive terminology has been proposed by Baker (2016b). The Natural History Museum holds a burrow cast made by the holotype of *Gryllotalpa vineae* and has made 3D models available (Baker and Broom 2015).

#### Baffles

Some tree crickets of the genus *Oecanthus* use baffles made of leaves to amplify their sound (Mhatre 2018).

**BaffleMaterial** https://vocab.audioblast.org/BaffleMaterial

### Hearing

Insects hear through modified tympanal organs, but they vary in their location on the body. In the Tettigoniidae the hearing organs are located on the foreleg tibia, whereas in the Acrididae they are located on the 1st abdominal segment. The hearing organ location property is used to record this information. The location of hearing organs has been summarised by Hoy and Fay (1998).

**HearingOrgan** https://vocab.audioblast.org/HearingOrgan

Currently one of 'SubgenualOrgan', 'TripartiteOrgan', 'Typanum'. A proposed controlled vocabularly is provided at https://vocab.audioblast.org/cv/hearing.

**HearingOrganLocation** https://vocab.audioblast.org/HearingOrganLocation

E.g. 'Tibia', 'Abdomen'. A proposed controlled vocabularly is provided at https://vocab.audioblast.org/cv/hol.

**Hearing Frequency** https://vocab.audioblast.org/HearingFrequency

The frequency range in kHz that the insect hears.

**Hearing Peak Frequency** https://vocab.audioblast.org/HearingPeakFrequency

The frequency (in KHz) at which the hearing is most sensitive.

## Data Models

The "bag of terms" approach used here leaves data models to the user, unless a future community effort is made towards standardisation. The models here provide some examples of how the terms may be used to describe sound production in insects. All of the examples here are taken from the literature.

### Basic facts about a call

"**A rapid succession of loud, sonorous chirps, almost always of three syllables. *Gryllus campestris*.**" (Bellman 1988: Table 6)

The term chirp is here deprecated following Broughton (1976) so the highest level of structure is the echeme sequence (the chirp is an echeme, the song is comprised of an echeme sequence).

"**Soft buzzing chirps of c. 1 sec. duration ('trrrrt'), separated by intervals of about equal length.** *Platycleis montana*." (Bellman 1988: Table 7)

This example is expanded to include a reference. The units of the Value column are defined above (as SI units) so there is no need to indicate them here.

"**Output energy in the 1996 specimen was centred at 124.8 kHz, with 126.5 and 122.2 kHz in each of the specimens collected in 2013 respectively, for an average of 124.5±2.17 kHz (n = 4, Fig. 7H)**" (Sarria-S et al. 2014: Table 8)

### Basic facts about morphology

"**The left and right files are equal in length and bear the same number of teeth. The right file has a mean length of 0.48±0.02mm (*N*=13) and the left file has a mean length of 0.48±0.03 mm (*N*=14). The number of teeth was 36±2 (*N*=13) on the right file and 36±3 (*N*=14) on the left file.**" (Dambach and Gras 1995: Table 9)

### Mutliple calls per taxon

"**The calling song of male *G. integer* consists of chirps with two or three sound pulses each (carrier frequency of approximately 4.2 kHz). ... By contrast to calling song, courtship song in *G. integer* consists of 4.2 kHz sound pulses interspersed with higher amplitude, higher frequency (13 kHz) single sound pulses.**" (Leonard and Hedrick (2010): Table 10)

### Hemisyllables

"***Artiotonus artius* ... At 24 ◦C, the song of this species is an un- broken wave train (a quite short very sinusoidal pulse) of 3.78 ± 0.14 ms duration (n = 7), produced by a single continuous closing stroke.**" (MONTEALEGRE-Z et al. 2011: Table 11).

This exaple also records the temperature, as many properties of insect songs are temperature dependant.

## Example implementation on BioAcoustica

As an example of the usage of this standardised terminology it has been implemented on the BioAcosutica website (Baker et al. 2015b). So far over 5,500 individual items of acoustic trait data have been added. BioAcoustica is bulit on top of the Scratchpads virtual research environment (VRE) (Smith et al. 2012). The terms proposed here are stored as a classification within the VRE, and a new bioacoustics_traits content type allows the linking of terms to species, temperature, sex and a published literature reference. An example from the user interface is given in Fig. 7

## Acoustic Ecological Interactions

The Global Biotic Interactions project (GloBI; Poelen et al. 2014) has driven the recent increase in the accessibility of ecological interaction data on the web.

**Figure 6.**
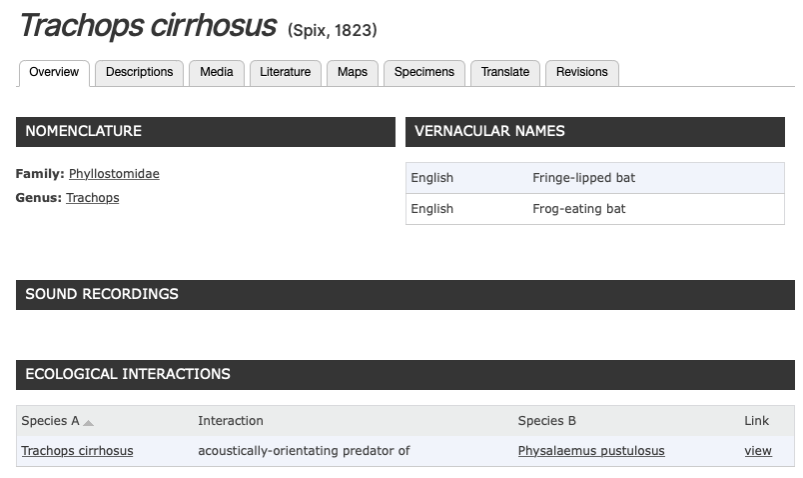
Acoustic ecological interaction implemented within the BioAcoustica platform.

**Figure 7.**
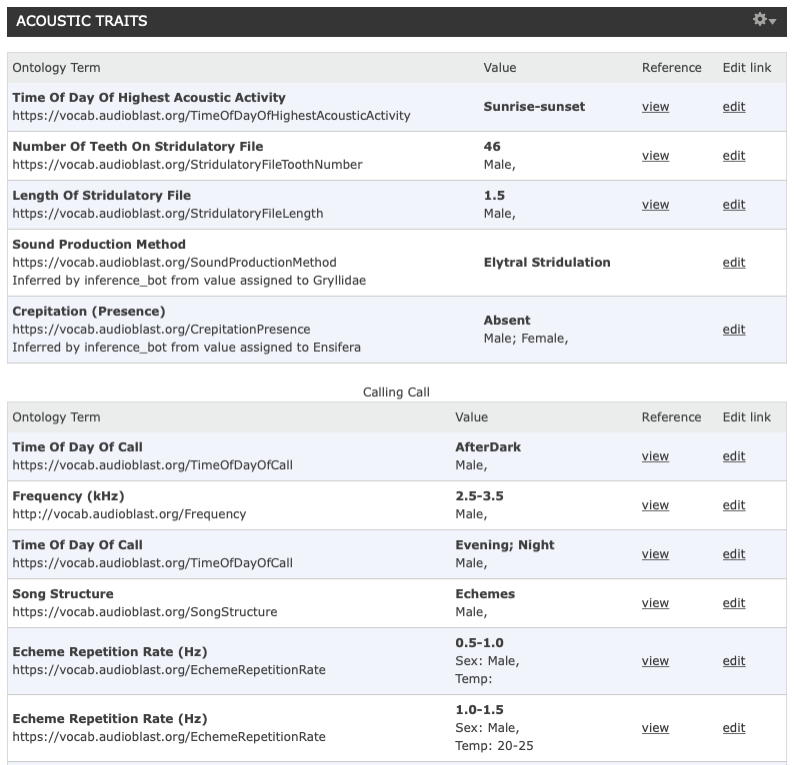
User interface for bioacoustic traits in the BioAcoustica platform.

The recent integration of ecological interactions into the Scratchpads VRE (Baker et al. 2019) has provided the opportunity for integration of some acoustic ecology terms into the BioAcoustica project (Fig. 6). While the current term list is small and based solely upon papers already in the BioAcoustica system, the future development of such a list seems appropriate to be done within the broader scope of the project outlined here. A list of terms is at https://vocab.audioblast.org/cv/ecoint.

## Discussion

The proposals made here address many of the issues the authors have faced in consolidating acoustic trait datasets for their own research purposes. It is anticipated that they will, in general, be of broader use, and with expansion, or modification be applicable to other scientists, or other taxonomic groups. As an example, it can reasonably be anticipated that terms relating to frequency and times of calls when applied to all acoustically active species in an area may provide useful information in the partition of the acoustic space between species.

The authors are willing, and interested in, collaborating with others to develop the proposed vocabulary for additional use cases. While this paper addresses only terminology associated with insects, every effort has been made to make the vocabulary itself taxon-neutral. Suggestions on improvements and additions are welcomed via GitHub (https://github.com/audioblast/vocabularies/issues) or by email.

## Future Work

Besides the general development of the terminology and associated vocabularies presented here, two main themes of work are currently planned.

The first is a centralised database of acoustic trait data that will harvest trait data from BioAcoustica and in future other data sources. This database will be searchable via a web-based API (Application Programming Interface) that will be used to power a website for end users and be accessible via an R package for scripted querying. This API will be publically available and documented for integration with other projects.

Work is underway on internationalisation of the vocabulary. This includes incorporating non-English terms into the controlled vocabularies and providing non-English translations of the term definitions.

## Figures and Tables

**Table 1.** Controlled vocabulary for types of calls in insects. The references for synonymous terms are only for indication of use. https://vocab.audioblast.org/cv/callType

Call Type	Notes
CallingSong	= Spontaneous song = Proclamation song = Advertisment song = Common song = Ordinary song = Solitary song = Usual song = Wonted song = Indifferent song
CongregationalSong	= Aggregating song
ResponseCall	
PrematingSong	Broader category than CourtshipSong, AgreeementSong, and JumpingSong
CourtshipSong	= Serenade song
AgreementSong	= Attraction song = Invitation call
JumpingSong	Shout of triumph (Dumortier 1963)
RivalryCall	= Aggressive song
PostcopulatoryCall	
DefensiveCall	= Alarm call = Protest sound = Disturbance song
FlightNoise	

**Table 2.** Controlled vocabulary for sound production method. https://vocab.audioblast.org/cv/spm

Method	Example Taxon	Notes
Stridulation		
Abdomino-alaryStridulation	Coleoptera (Wessel 2006)	
Abdomino-elytralStridulation	Coleoptera (Wessel 2006)	
Abdomino-femoralStridulation	Coleoptera (Wessel 2006)	
Alary-abdominalStridulation	Coleoptera (Wessel 2006)	
Alary-elytralStridualtion	Coleoptera (Wessel 2006)	
AntennalStridulation	Phylliidae (Delfosse 1999)	
Coxo-metasternalStridulation	Coleoptera (Wessel 2006)	
Cranio-prothoracaicStridulation	Coleoptera (Wessel 2006)	
ElytralStridulation	Ensifera (Ragge and Reynolds 1998)	
Elytro-abdominalStridulation	Coleoptera (Wessel 2006)	
Elyto-femoralStridulation	Coleoptera (Wessel 2006) Orthoptera (Ragge and Reynolds 1998)	Otte (1972) makes a distinction between Ordinary stridulation and Vibratory stridulation, however the only difference appears to be the speed of the movement and not the production method.
FemoralStridulation	Coleoptera (Wessel 2006)	
Maxillo-mandibularStridulation	Coleoptera (Wessel 2006)	
MesothoracicScutellum-elytralStridulation	Cicadidae (Moulds 2005)	
Mesonoto-elytralStridulation	Cicadidae (Moulds 2005)	
Mesonoto-pronotalStridulation	Coleoptera (Wessel 2006)	
Pronoto-femoralStridulation	Coleoptera (Wessel 2006)	
Prosterno-mesosternalStridulation	Coleoptera (Wessel 2006)	
Crepitation	Acrididae (Lorier et al. 2012)	
Percussion		
Elytro-tibialPercussion	*Stethophyma grossum*	The form of elytro-femoral stridulation in this species appears to be unique. The hind tibia are flicked at the flexed fore wing (Ragge and Reynolds 1998). This behaviour seems to be consistent with the Ticking described by Otte (1972).
Hindleg-substratePercussion	*Meconema* (Benton 2012)	
Head-susbsratePercussion	Termitoidea (Connétable et al. 1999)	
Vibration		
WingVibration	*Heteropteryx* (Delfosse 1999)	
FluidExpulsion		
PharyngealAirExpulsion	Sphingidae (Brehm et al. 2015)	
SpiracularAirExpulsion	Gromphadorhinini (Clark and Moore 1995)	
Tremulation		
AbdominalTremulation	Coleoptera (Shestakov and Kasparson 2019)	
BodyTremulation	Orthoptera (Morris 1980)	
Tymbalisation	Cicadidae (Boulard 2013)	

**Table 3.** Controlled vocabulary for call components. https://vocab.audioblast.org/cv/components

**Component**	**Related properties**
Pulse	PulseDuration PulseInterval PulsePeriod PulseRepetitionRate
Syllable	SyllableDuration SyllableInterval SyllablePeriod SyllableRepetitonRate
	SyllableDurationInEcheme SyllableDurationFinal SyllableDurationFirst SyllableDurationIsolatedSyllable SyllablePeriodIsolatedSyllable SyllableRepetitionRateInEcheme
	PulsesPerSyllable
Diplosyllable	
Hemisyllable ClosingHemisyllable OpeningHemisyllable	HemisyllableDuration
	HemisyllableDurationDownstroke HemisyllableDurationFinal HemisyllableDurationFirst HemisyllableDurationUpstroke
Echeme	EchemeDuration EchemeInterval EchemePeriod EchemeRepetitionRate
	EchemeDurationFirstEcheme EchemeDurationFinalEcheme
	SyllablesPerEcheme
EchemeSequence	EchemeSequenceDuration EchemeSequenceInterval
	EchemesPerEchemeSequence
Call	CallDuration CallInterval

**Table 4.** Controlled vocabulary for mate location method. https://vocab.audioblast.org/cv/mlm

**Mate-location Method**
MalePhonotaxis
FemalePhonotaxis
MaleAndFemalePhonotaxis
MaleOrFemalePhonotaxis

**Table 5.** Controlled vocabulary for male behaviour modifications to conspecfic Calling Song. https://vocab.audioblast.org/cv/maleres

**Male resposne to conspecific song**
PhysicalSpacing
Chorusing
SynchronousChorusing
AlternateChorusing
UnsychronousChorusing

**Table 6.** Coding for *Gryllus campestris* from the key in Bellman (1988)

Species	Property	Value
*Gryllus campestris*	Call structure	EchemeSequence
*Gryllus campestris*	Syllables per echeme	3

**Table 7.** Coding for *Platycleis montana* from the key in Bellman (1988).

Species	Property	Value	Reference
*Platycleis montana*	CallStructure	EchemeSequence	Bellman (1988)
*Platycleis montana*	EchemeDuration	1	Bellman (1988)
*Platycleis montana*	EchemeInterval	1	Bellman (1988)

**Table 8.** Coding for frequency of *Supersonus piercei* from Sarria-S et al. (2014).

Species	Property	Value	Ref
*Supersonus piercei*	CentreFrequency	124.5±2.17	Sarria-S et al., 2014

**Table 9.** Coding for morphological features of *Cycloptiloides canariensis* from Dambach and Gras (1995).

Species	Property	Value	Reference
*Cycloptiloides canariensis*	StridulatoryFileLength	0.48±0.03	Dambach and Gras (1995)
*Cycloptiloides canariensis*	StridulatoryFileToothNumber	36±3	Dambach and Gras (1995)

**Table 10.** Coding for different songs in *Gryllus integer* from Leonard and Hedrick (2010).

**Species**	**CallType**	**Property**	**Value**	**Reference**
*Gryllus integer*	CallingSong	PeakFrequency	4.2	Leonard & Hedrick (2010)
*Gryllus integer*	CourtshipSong	PeakFrequency	4.2; 13	Leonard & Hedrick (2010)

**Table 11.** Coding for song structure from MONTEALEGRE-Z et al. (2011).

Species	Property	Value	Temperature	Reference
*Artiotonus atius*	CallStructure	ClosingHemisyllable	24	Montealegre-Z et al, 2011

## Hosting institution

University of York
